# A Systems Theory of Mental Health in Recreational Sport

**DOI:** 10.3390/ijerph192114244

**Published:** 2022-10-31

**Authors:** Stewart A. Vella, Matthew J. Schweickle, Jordan Sutcliffe, Caitlin Liddelow, Christian Swann

**Affiliations:** 1 School of Psychology, University of Wollongong, Wollongong, NSW 2522, Australia; 2 Global Alliance for Mental Health and Sport, University of Wollongong, Wollongong, NSW 2500, Australia; 3 Physical Activity, Sport, and Exercise Research Theme, Faculty of Health, Southern Cross University, Coffs Harbour, NSW 2450, Australia

**Keywords:** wellbeing, psychological safety, coaching, policy, public health

## Abstract

The focus on mental health outcomes in sport, including the ways in which mental health can be protected and promoted, has become a major international priority for all sports, including the recreational sports system. The aim of this paper is to outline a systems theory of mental health care and promotion that is specific to needs of the recreational sport system so that context-specific, effective policies, interventions, and models of care can be articulated and tested. Based on general systems theory, we offer a preliminary theory and accompanying postulates that outline the general principles that explain mental health in recreational sports. Namely: one of the purposes of the recreational sport system is to protect and promote the mental health of all involved; the recreational sport system is comprised of components (individuals, groups, organisations, communities, society); the role and function of those components vary by context; the purpose of the system is achieved through coordinated and reciprocal relationships between its components; and, the system should be regulated from within to ensure that mental health is protected and promoted. We have also outlined the ways in which the subcomponents of the system interact, their potential influence on mental health in recreational sports, and the ways in which they may be regulated. This information provides a theoretical foundation upon which research, programming, and policy can be based to protect and promote the mental health of all involved in recreational sports.

## 1. Mental Health in Recreational Sport

Organised sports are one of the most popular forms of leisure time physical activity worldwide. In fact, at least half of all children and adolescents participate in organised sports in most developed countries globally, while these rates are much higher in high-income countries such as Canada, Denmark, and New Zealand [1]. Among adults, participation rates are substantially lower than those among children and adolescents. Nevertheless, many adults participate in organised sports gloabally [2]. The vast majority of sport participation occurs within organised recreational sports where the primary purposes of participation are enjoyment and health [3]. Indeed, there is strong evidence to suggest that participation in organised sports is beneficial for health and wellbeing. For example, participation in football (soccer) is beneficial for cardiovascular health, including improved aerobic fitness and cardiovascular function, as well as reductions in adiposity [4]. There is also evidence that sport participation is associated with psychosocial benefits such a better mental health and wellbeing, better social interactions, and increased self-esteem [5,6].

In recent years, the focus on mental health outcomes in sport has proliferated across recreational and elite sport. For example, a review of local sport organisations websites in Australia found that almost 60% had mentioned mental health as associated with participation in the sport, including mental health benefits, the prevention of mental health problems, and the promotion of mental health websites [7]. Academic interest in the topic has followed suit, with a plethora of reviews published recently on topics such as: the mental health of elite athletes [8]; mental health interventions in non-elite sport [9]; psychotherapy for mental health symptoms and disorders in elite athletes [10]; mental health literacy interventions in sport [11]; and associations between depression and anxiety symptoms and participation in youth sports [12]. Furthermore, best practice guidelines for the policy, practice, and research of sport psychology in relation to mental health have also been put forward [13]. Mental health in sport, therefore, represents both an important, and rapidly growing, field of research.

To begin to address mental health in sport, at least 13 distinct position and consensus statements have been published [14]. These statements have been developed and endorsed by organisations including the International Olympic Committee [15], International Society for Sport Psychology [16], and the National Collegiate Athletics Association [17], among others. These statements have also been the subject of their own review [14]. That review found that both the quality and the consistency of those statements was low, most notably pertaining to the rigor with which they were developed. In addition, only one of those statements was addressed at recreational athletes, and specifically high school athletes [18]. As such, there has been a call to expand mental health policy initiatives to recreational sports [19], which account for the vast majority of sport participants worldwide. This is symptomatic of a general trend whereby recreational sport has typically received far less empirical and policy attention guarding mental health when compared to elite level sports.

Whilst the existing position statements incorporate a wide array of stakeholders, these statements lack any notable theoretical underpinnings. A theoretical foundation is fundamental to accounting for the reciprocal and dynamic relationships in which sport takes place. As a case in point, Vella et al. [14] have highlighted contradictions across these position statements precisely because they do not deal adequately with the interconnectedness of individuals and organisations in sport. To move forward, theoretical frameworks of mental health that are specific to the sport system are urgently needed to inform effective mental health protection and promotion.

The aim of this paper is to outline a systems theory of mental health care and promotion that is specific to needs of the recreational sport system so that context-specific, effective policies, interventions, and models of care can be articulated and tested. In doing so, we aim to facilitate an integrated, systemic, and interdisciplinary understanding of the provision of mental health care and promotion in recreational sport. Based on the principles of general systems theory [20], we will define the system of recreational sport, as well as the components of the system as they relate to mental health, give an overview of how those components of the system relate to one another, and in so doing, describe the influence that the system (through its components) has on mental health of all involved in recreational sport. The benefits of a systems theory of mental health specific to recreational sport include: the facilitation of scientific progress in the field such that researchers, practitioners, and policy makers can move forward in an integrated and systematic manner; promote an interdisciplinary understanding of mental health in recreational sport upon which research and policy can be based; facilitate more impactful research that can influence policy and practice; illuminate the various ways in which program designers and administrators can influence mental health in recreational sport; inform the development of high quality recreational sport programs in regard to mental health; and, shape the design and delivery of mental health care within the recreational sport system.

## 2. Review of Existing Approaches

One model that has been put forward to inform sport psychology practice in relation to athlete mental health is the optimisation intervention model, which is aimed at promoting both athlete performance and mental health, and is grounded in cognitive behaviour therapy [21]. This model provides an adaptation of CBT to accommodate athlete performance. Whilst this model is valuable in informing sport psychology practice in elite sports where the focus is on optimising athletic performance [3], it is difficult to apply within recreational sports where one-on-one practice is unlikely to occur, where performance is not always the primary goal, and where policy makers may be actively seeking to shape the recreational sport environment for mental health promotion rather than providing sport psychology services to individual athletes.

A model of mental health care in elite sport has been put forward by Van Slingerland et al. [22] in which the authors articulated an intake process and care pathway for elite athletes, as well as a collaborative care model. Importantly, the collaborative care model considers the various individuals and subsystems which surround elite athletes, including mental performance consultants, psychologists, dieticians, family members, and the sports organisation (among others). However, the model was designed specifically for elite athletes, and is not (and was not meant to be) generalisable to recreational sport.

Alternatively, Purcell et al. [23] articulated a comprehensive framework and model of care to support and respond to the mental health needs of elite athletes. The framework is based on Ecological Systems Theory [24] which provides for an understanding of an elite athlete within a multi-layered system of influences on their mental health which include interpersonal, organisational, sport system, and societal influences. The benefit of using such a theory is that it can allow for the interconnectedness of people and organisations, and also allow for intervention at the individual, sporting, and/or organisational levels. This model, however, is also developed specifically for elite athletes, and is not generalisable to recreational sports.

Elite athletes are embedded within structured support systems which have been modelled by Van Slingerland et al. [22] and Purcell et al. [23], however the resources within such systems are not afforded to recreational sports, which are typically run by volunteers with little time, few resources, inadequate training (especially in mental health), and have far less contact time with participants [25]. For this reason alone, very different models of mental health in recreational sport are needed to those provided for elite athletes. Other reasons also include vast differences in the primary purpose of participation between elite and recreational sport [3], and differences in the psychological stressors experienced by elite and recreational athletes [19]. In the sections below, we outline a theory that can fill this void.

Recently, there has been a trend toward the use of general systems theory in attempting to understand, explain, and inform the practice of improving psychosocial outcomes for people who participate in recreational sports by understanding the system as a whole. Notably, Dorsch et al. [26] provided an integrated understanding of the youth sport system based on general systems theory. They defined the youth sport system as ‘the set of interdependent persons (i.e., parents, siblings, peers, and coaches) and contexts (i.e., organizations, communities, and societies) that have the potential to influence or be influenced by an athlete’s behaviors, attitudes, experiences, and outcomes in youth sport’ (p. 2). They further defined three subsystems within youth sport, including: the family subsystem which incorporates athletes, siblings, and parents; the team subsystem, which includes the athlete, peers, and coach; and the environmental subsystem, which includes organisations, communities, and societies. In so doing, Dorsch et al. (2020) [26] attempted to move the field towards a ‘systemic and interdisciplinary understanding’ (p. 11) of the ways in which interdependent actors, groups, and organisations, and environmental factors interact over time to influence young athletes.

Extending on this work, Dorsch et al. [27] outlined systems theory as “a dynamic framework that can be used by clinical and/or counselling sport psychologists in the design and delivery of interventions in organized sport” (p. 243) at all levels, and notably including recreational sport. The authors made the point that clinical and counselling interventions in sport should consider the collective nature of the sport system, which includes the subsystems of the family, team, and environment. In so doing, lessons are drawn for practitioners who work in applied settings across the developmental spectrum including youth athletes, high school athletes, intercollegiate athletes, and elite athletes. It is held that by viewing the athlete as situated within a broader system and subsystems that are mutually influential, a holistic approach to applied practice can be pursued in order to enhance athlete performance and wellbeing. The authors concluded that “understanding the systemic influences that shape these outcomes is essential to supporting athletes’ coping mechanisms, social skills, and relationship dynamics that can ultimately lead to enhanced outcomes for athletes as well as families and teams in sport” (p. 254).

Taking an empirical approach, Whitley et al. [28] applied systems theory in the more specific context of facilitating positive youth development of disadvantaged youth and those who have experienced trauma. The authors propose a model of the optimal sporting system through which youth may engage in sports. In line with the basic principles of general systems theory—although the theory is not referred to specifically—the model places the youth within an integrated system of mutually influential subsystems to achieve the purpose of positive youth development. Those factors and subsystems articulated within the model include an intra-individual process (of embodiment where youth can reconnect with their physical and emotional selves). In turn, being an athlete was both influenced by, and has an influence on, one’s environment. The authors articulately made this point in stating that “data revealed that ‘being an athlete’ changed the way those in the environment treated youth, thus prompting a more agentic reciprocal determinism of individual and environmental influences” (p. 121). The athlete is also situated in a developmentally focussed sport program, which in turn, can influence the ways in which positive contributions to the community can be understood and enacted. This point is reinforced and extended by Massey et al. [29] who outlined the ways in which individuals and sports programs can effect meaningful change at a community and societal level via a systems theory lens, with a focus on the field of sport for development and peace. The conclusion to be drawn from this line of inquiry is the strength of the subsystems which underpin the ‘whole’ sporting system, which offer the opportunity for the design of sports programs to optimise both individual and community level outcomes.

## 3. Overview of General Systems Theory

General systems theory at its most fundamental level represents a collection of principles that apply to all systems [20]. The theory is defined by exploration of the system as a whole, on the premise that an understanding of the whole is only possible through an understanding of its constituent components and the interrelations among them, and with the environment [20]. A system can be defined as a set of interacting components that form an integrated whole which is intended to perform a specific function. Indeed, the purpose of the system is the ‘reason for its existence and the starting point for measuring its success. The purpose of a system is what is does’ ([30] p. 57). For example, the recreational sporting system is comprised of multiple organisations, entities, and persons who interact for the purpose of providing recreational sports programs. Systems such as these, which have both structure (e.g., organisations, coaches, athletes) and activity (delivery of sport programs) are generally considered dynamic systems.

There are several properties of a system that are generally considered to constitute general systems theory [30]. First, a system (e.g., a sports system) is comprised of components that are interrelated and interdependent. Second, those elements combine within a system to produce a ‘whole’ (e.g., recreational sport), which has properties that are not evident in the components alone. Third, the interaction of the system components results in some end goal (such as the delivery of sport programs). Fourth, those interrelated components of the system must be regulated in order to achieve that goal, where deviations are detected and corrected. Fifth, corrections to the system or its components (e.g., individuals, organisations) are made via the interactions between the components, which are necessary for transformation. Sixth, the whole system (e.g., sport system) is comprised of smaller components (e.g., a sports club or sports league) which are nested within the larger system (e.g., national or state governing body), creating a hierarchy. Seventh, within complex systems each component of the system performs specialised functions which differentiate them from the other components (e.g., national sports organisations, regional associations, sports clubs, referees, athletes, etc.), and those functions are governed by a set of rules or roles that are negotiated over time. Finally, systems can have multiple ways to achieve the same goal given different conditions (convergence; e.g., there are multiple ways of delivering sports programs), or from a given starting position, can achieve different, mutually exclusive objectives (divergence; e.g., provision of sport programming and facilitating participant mental health). It is our aim to apply these meta-theoretical principles of general systems theory within a theory of mental health in recreational sport.

## 4. Systems Theory to Explain Mental Health in Recreational Sport

We define the recreational sport system as the set of interdependent persons (i.e., athletes, parents, siblings, peers, coaches, and volunteers), groups (i.e., teams), and organisations (i.e., clubs, leagues, organisations, governing bodies) that are involved in the provision and participation of recreational sports programs. We further define *recreational* sports programs as those where the primary purpose of the program is participation, rather than performance. According to the Developmental Model of Sports Participation, recreational sport participation from ages 6–12 years is characterised by a focus on deliberate play with the objective to maximise inherent enjoyment [3]. From the age of 13 years and up, recreational sports programs are characterised by the primary goals of enjoyment and health, and include both deliberate play and deliberate practice, with a greater emphasis on deliberate play. The recreational sports programs are differentiated from elite sports programs where the purpose of participation is optimal performance [3]. Swann et al. [31] provided guidelines around the quantification of elite sports, whereby recreational sports can be assumed to include all non-elite programs. Operationalising the taxonomy provided by Swann et al. [31], recreational sports are those where the athletes are performing at a level that is: lower than a regional level; lower than a university or collegiate level; not semi-professional or professional; and equivalent to something lower than the 4th tier league or tournament available.

In defining the recreational sports system, we borrow some of the key components of the definition provided by Dorsch et al. [26] regarding the youth sport system—the most popular form of recreational sport [1,2]—most notably, the notion and conceptualisation of the various subsystems within recreational sport, and the assumption that those subsystems are interconnected in dynamic and reciprocally influential ways that produce a set of outcomes for individuals. However, where Dorsch et al. [26] place the athlete at the centre of the youth sport system, we deliberately broaden the ‘end user’ of the recreational sport system to all those who are involved within the system on the assumption that the mental health of athletes, coaches, volunteers (and all others involved in the system) should be considered. In other words, the whole system should be designed and implemented in a way that protects and promotes the mental health of everybody involved. For example, coaches, officials (i.e., referees and umpires), and volunteers (e.g., committee members, administrators) may undertake those roles as an end in and of itself, and therefore the purpose of the system is as much to provide opportunities for those people to participate as much as it is for the participation of athletes. This is consistent with recent calls to focus on the mental health of all stakeholders in recreational sport including officials [32] and youth sport parents [33].

In applying the principles of general systems theory to an understanding of mental health in recreational sport, we articulate five key postulates:

***Postulate 1***: The primary purpose of the recreational sports system is the provision and delivery of sports programs for fun and enjoyment. However, in line with the principle of divergence, a system can achieve different, mutually exclusive objectives. One of these objectives for the recreational sport system is to protect and promote the mental health of individuals involved within the system.

***Postulate 2***: The system is made of components which perform specialised functions in order to ensure the successful delivery of recreational sport programs. Those components and subsystems include individuals (e.g., athletes, parents, siblings, coaches, and volunteers), groups (e.g., teams, age groups), and organisations (e.g., clubs, leagues, governing bodies of varying levels), all of whom operate within, and exert an influence on, their community and society.

***Postulate 3***: Components of the system, as well as the functions of these components, vary by context (e.g., sport, state, country). In line with the principle of convergence, variations in components and their functions represent different ways to achieve the purpose of recreational sport programs.

***Postulate 4***: The objectives of the recreational sport system—for example, to provide sport programs for fun and enjoyment, and to protect and promote the mental health of individuals—are achieved through the coordinated and reciprocal relationships between the components of the system.

***Postulate 5***: The components of the system must be regulated in the undertaking of their specialised functions so that deviations can be detected and corrected. In this case, the delivery of recreational sports programs must be regulated to ensure that no psychological harm is done. While the system operates in a society and community with certain expectations of the function of that system, regulation is necessary from within the system through explicit processes of self-regulation (e.g., mandatory training for coaches, guidelines on the treatment of officials, etc).

**Figure 1:**
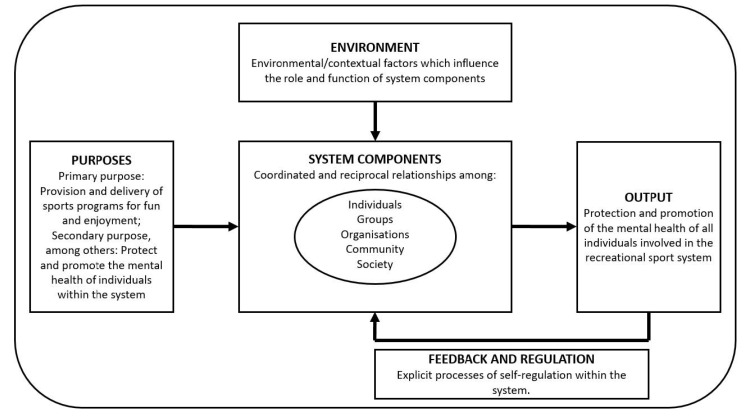
An overview of the theoretical postulates of the theory of mental health in recreational sport.

Importantly, we acknowledge that the nuances of system components, including their interrelations, the rules and roles that govern their functioning, and cultural understandings of mental health, vary meaningfully by context. As such, the strict and specific application of principles to all recreational sports systems are unlikely to be helpful. However, we attempt here to apply some very general and universal principles to the recreational sports system. In the following sections we aim to define the recreational sport system as it functions in relation to mental health of all participants, including an outline of the major components of that system, their roles and functions, the ways in which they interrelate, and the ways in which they may be regulated. We do not wish to replace current efforts to integrate an understanding of the youth sport system [26], or applied psychological practice within sports [27]. It is our goal to leverage these important efforts, and apply the principles within them specifically to mental health in recreational sport. Indeed, this sole focus on mental health requires its own nuance in the interrelations and roles of individuals, components, and environment in the pursuit of the protection and promotion of mental health for all people involved in recreational sports programs. An overview of the theoretical postulates is given in Figure 1. The system components are explained in more detail below.

## 5. System Components

### 5.1. Society

It is broadly recognised that the design and implementation of recreational sports programs and sports-based mental health interventions are largely influenced by the cultures [21,27] and societal norms (e.g., [26]) in which the club exists. Dorsch et al. [26] note that societies influence the meaning, design, and delivery of recreational sports programs via societal norms. For example, societal expectations around the role of sports clubs can govern the types and breadth of care that are provided, as well as the ways in which that care is enacted. For example, the Good Sports program in Australia [34] helps community sports clubs to comply with their legal requirements for the responsible service of alcohol. The Good Sports program has over reached over 9500 clubs and a total of over 2.26 million athletes and their families nationally [35], and includes resources, training, guidance and an accreditation system to govern alcohol service within those clubs. In this way, society (in the form of laws and national, government-funded organisations) have had a meaningful influence on the way that care is provided to participants of recreational sports. However, Dorsch et al. [26] note that the influence of society goes beyond policy, and also includes values, traditions, sponsorship, and socialisation. They note that “Youth sport is generally designed and delivered in ways that reflect and reaffirm the traditions and values that are important in society” (p. 6).

There are also ways in which recreational sporting programs can influence society more broadly. Taking a systems theory approach, Massey et al. [29] have outlined the ways in which sport for development and peace (SFDP) programs can have a meaningful impact on society. The authors summarised a broad literature including the potential benefits of SFDP programs at a societal level, including increased health, development, education, gender equity and female empowerment, inclusion of people with disabilities, social inclusion, conflict resolution, and peace [29]. For example, participation in football (soccer) has demonstrated health benefits [4], and given the proportion of the people who participate in football (soccer), this is likely to have benefits to society, including lower burden on the health care system and lower health care resource use.

It is also worth noting that the changing public perceptions around mental health in sport can influence the care that is provided in recreational sports. For example, the Rugby League World Cup 2021 has adopted a mental fitness charter, and have undertaken to deliver mental health literacy programming to over 8000 athletes, parents, and coaches within community rugby league clubs in the United Kingdom [36]. On the back of such initiatives, calls have been made for mental health guidelines to be put in place specifically for recreational sports [19]. These calls have been precipitated by general increases in societal levels of mental health literacy, which are likely to be reflected in the design and delivery of recreational sports programs. In turn, the provision of mental health programs within recreational sports can increase mental health literacy and wellbeing [37], which can have a downstream impact on health care costs and resource use that is cost-effective [38].

Moving forward, shifting societal values around mental health will continue to shift societal expectations and norms around the mental health care provided to those within recreational sporting programs. As noted by Dorsch et al. [26], these values and norms can be expressed in positive or negative ways that can lead to either mental health benefits or mental health detriments. Perhaps the most effective regulation initiatives are those in the form of public law and policy that mandate changes within the sport system that lead to improved care and outcomes for everybody involved. Furthermore, the provision of mental health resources at a societal level (e.g., funding for mental health services, public mental health literacy initiatives, charitable and non-government organisations) can influence the type and breadth of care available within recreational sports. Finally, formal articulation or endorsement of mental health guidelines for recreational sports by governmental bodies can help to regulate the care provided to individuals within the recreational sports system.

### 5.2. Communities

Dorsch et al. [26] noted the intimate and reciprocal links between youth sport programs and the communities in which they are situated. For example, they noted that “communities establish and fortify the norms associated with sport participation while also providing support at the group level and fostering a sense of belongingness among individuals” (p. 6). They also noted that in the United States (as well as more broadly) organised sports can be synonymous with community through Little Leagues, Parks and Recreation Departments, and can also be important components of a community in highly formalised and less formalised ways [39]. For example, community resources such as small-to-medium enterprises may sponsor recreational sports teams or leagues. Importantly, Wicker and Breuer [40] have shown how the economic and financial situation of the community influences the human and financial resources of community sports clubs. For example, in communities where unemployment is high, community sports clubs have less volunteer problems, whilst within larger communities, sports clubs have greater financial and facility problems.

Community sports clubs can have a meaningful impact on the communities in which they reside. For example, community sporting clubs can fulfil social and health-related obligations which will have a meaningful impact on the community, including the creation of social capital, provision of opportunities for physical activity, and the promotion of health [41]. To illustrate this point, the International Society for Physical Activity have endorsed organised sports as one of the eight ‘investments that work’ to increase physical activity [42]. In addition, community sports clubs, as well as their components and constituent parts (i.e., athletes, coaches, parents, teams) can influence and promote the development of young people to become active and meaningful contributors to their community [43]. As such, the recreational sports systems can provide meaningful tangible and intangible benefits to the communities in which they are embedded through individual, group, and societal level change.

With specific reference to mental health, communities exert an influence on the recreational sports system at various levels and through broad means. Kokko [44] has modelled such influences as including social, cultural, economic, and environmental determinants of health within sports clubs, and as including micro-level systems such as coach-athlete relationships, meso-level systems such as the sports club board/committee, and macro-level systems such as the sports club as a whole. Social determinants at a community level include the expectations placed on clubs and coaches to articulate and implement mental health care policies within the club. Such views are captured within a series of studies on the role of sports clubs, coaches, and parents on mental health promotion within community sporting clubs [45,46,47,48]. Economic determinants include the resources such as time and money available for mental health promotion [44]. For example, community resources such as access to affordable, accredited mental health care systems and providers will influence how recreational sports systems provide mental health care. Where affordable mental health care can be readily accessed, sports clubs may engage in partnerships with qualified mental health care providers to present programs or occupy a formal role within the club (e.g., mental health first aid officer), or clubs may simply refer individuals to such providers. In the absence of such providers, however, sports clubs have meaningfully less options for the provision of appropriate and qualified care.

Alternatively, charitable organisations are also actively working in the mental health in sport space and can also offer valuable community-level resources. For example, on the back of the Rugby League World Cup 2021, the Rugby League Cares Foundation and Movember have funded and resourced the delivery of mental health literacy and resilience programs to young rugby league participants across the United Kingdom [36]. In Australia, the Australian Football League commissioned a rapid review of the mental health programs currently on offer in Australia, with a large number of community and charitable organisations offering programs in this space—although only a small number were found to be of sufficient quality to warrant endorsement by the governing body [49]. This rapid review is an example of how national sports organisations can influence the recreational sporting system by providing a list of available and appropriate programs that have some form of recommendation from the governing body. Such guidance from national sports organisations can help community sports clubs identify the community-level resources available to them that are of sufficient quality, and removes the need for such expertise to be developed within community sporting clubs to make decisions regarding the likely quality and appropriateness of mental health programs.

Similarly, governing bodies may help to regulate the community programs and/or community providers that work within the recreational sports system. By establishing formal relationships at a national or regional level, governing bodies can provide access to the community mental health care providers that are required by sports clubs and the individuals within those clubs. Further, governing bodies may also regulate the programs and providers that work within the system by providing formal guidance, approval, or endorsement of those programs which meet minimum standards of quality and evidence and are appropriate for delivery within recreational sport. As such, all levels of organisations clearly have a meaningful impact on the individuals who participate, volunteer, or administrate within community sporting clubs and the recreational sports system more broadly.

### 5.3. Organisations

There are a number of organisations involved in the recreational sports system. These include national sports organisations and governing bodies, state and regional sports organisations or leagues, community sports clubs, and schools and school districts, among others. While most or all of these organisations can function independently, they are all immersed within a recreational sport system where they influence, and are influenced by, the other organisations within that system. For example, sports clubs may compete in a regional league which is governed by a regional sports association/organisation, which is governed by a state sport organisation, which is in turn governed by a national sports organisation. The individuals who contribute to the implementation of recreational sports programs within community sports clubs can also exert an influence on the way in which recreational sports programs are implemented [26]. Similarly, a sports club or group of clubs exert influence on a regional league, and so on as the influence moves upwards in the chain.

For these reasons, Dorsch et al. [26] have placed organisations at the centre of the youth sport system. As such, organisations can often serve as the intermediary between societal and policy-level influences, and the individuals who are embedded within an organisation [26]. Community level sporting clubs, for example, are intermediaries in the sense that they are often responsible for the implementation of sport- and health-policies that are written or endorsed by governmental bodies or national/state sporting organisations (e.g., [50]). In this sense, it is important to distinguish between the varying levels of organisations which exist within the recreational sporting system, including the distinct roles that they may play. National sports organisations may be responsible for endorsing or writing policies, which are implemented by community sporting clubs, and where compliance is overseen or regulated by a regional level organisation.

In line with the above assumptions, there are various levels of organisations within the recreational sports system that exert reciprocal influence on each other, with a resultant impact on the mental health of all individuals who are involved in the system. For example, community sports clubs exert an influence of athlete mental health [46,47,48], parental mental health [33], and the mental health of officials [51]. In this sense, community sports clubs are a direct provider of sports programs which influence the mental health of individuals within those clubs through the uptake and implementation of policies, and also through the ways in which the sports programs are designed and implemented. For example, evidence-based coach training may be mandatory for all recreational sports coaches—with training provided and mandated by a national sports organisation, delivered by regional sports organisations (e.g., through coach educators), and enforced by community sporting clubs. Community sports clubs may also be a conduit for the delivery of evidence-based mental health programs for individuals within the clubs (such as those provided by charitable organisations or mental health care providers) which may be endorsed or mandated from governing organisations [49]. Community sports clubs may also provide referrals to qualified mental health providers through formal channels established within the club, or at higher levels (e.g., a referral partnership between a national sports organisation and a national mental health care provider).

Notably, the regulation of organisations and the individuals within them may also occur via other organisations within the system. In line with postulate 5 (i.e., the regulation of the components of the system), while the system operates in a society and community with certain expectations on the role of mental health care in recreational sport, regulation is nonetheless necessary from within the system through explicit processes of monitoring and oversight. Such monitoring and oversight can be undertaken by community sports clubs themselves (e.g., monitoring the qualifications and practices of coaches, and monitoring spectator behaviour), regional/state level organisations (e.g., through the monitoring of policy adoption, provision of coach education), and/or by national sports organisations (e.g., oversight of policy adoption, adjudications on deviations from policy).

### 5.4. Individuals

There are a number of individuals that exist within the recreational sports system for the purpose of delivering and participating in a recreational sport program. Most notably, this includes athletes, parents, coaches, officials, and administrators. While most of these individuals contribute to the sports system for the purpose of delivering the sport program *to athletes*, they also include roles such as coaches, officials, and administrators which can be undertaken as an end in and of itself. For this reason, and in line with postulate 1, we define one of the aims of the recreational sport system to protect and promote the mental health of those individuals involved within the system—including athletes, parents, coaches, officials, and administrators. Furthermore, these individuals are often nested within components that commonly include teams, family units, and age groups. While the most proximal influences include the athletic triad of athlete, coach, and parent, the influence of peers/teammates is also significant [26]. However, the nature of such influences changes over time, with parents and coaches being most influential on athletes at younger age groups, while peers are most influential from adolescence onwards [52]. Moreover, the sphere of influence that individuals may have on one another may also change over time. For example, coach influence on athlete effort and enjoyment is strongest during childhood, while their influence on perceptions of competence is strongest during adolescence [52]. This is also true of outcomes relevant to mental health. For example, peer punishment is associated with anxiety during childhood, but not adolescence [52].

Broadly, there have been calls to protect the mental health of all of these stakeholders [19]. For example, the protection of athlete mental health has been the focus of a large program of research in recreational sport (e.g., [37,45,46,47,48,53,54]). More recently, focus has turned to protecting the mental health of officials [32,51]. Similarly, there has been a recent focus on protecting and promoting the mental health of parents in youth sports [33]. Many of these lines of research have focussed on the ways in which other individuals within the system influence mental health at the individual level. For example, Brown et al. [45] investigated the ways in which parents and coaches can interact to promote athlete mental health in youth sports. Tingle et al. [51] noted that aggression towards officials negatively influences their mental health. Further, Sutcliffe et al. [33] noted how social support from other parents within the youth sport system can help to enhance and protect the mental health of youth sport parents.

Pertinently, many authors have noted the ways in which the recreational sport system can influence individual behaviour with regard to individual mental health. Tingle et al. [51] describe in detail the gender issues that result in poor mental health among female officials, which can be exacerbated by the actions of individuals such as coaches—an example of the ways in which different levels of the system interact to influence individual mental health. In addition, the authors also noted systemic issues such as the inability to address mental health stigma in the sport system, and a lack of organisational support and resources for the mental health of female officials [51]. Sutcliffe et al. [33] modelled this process more explicitly and postulated that the sport context and social actors influence short term outcomes such as interpersonal interactions and resource expenditure, which in turn influence the mental health and wellbeing of parents within the youth sport system. Another example can be drawn from Wynters et al. [55] whereby adolescent sport participants may encourage help seeking among their teammates for mental health problems—something which can be made much easier through sports systems with, for example, formal partnerships between sports clubs/organisations and mental health providers, and endorsed mental health literacy programs which are available for community sporting clubs. Finally, the existence and quality of mental health training offered to coaches, as well as organisational support for coaches within the recreational sports system is likely to influence the ways in which coaches will act to support athlete mental health [46,56]. Thus, ways in which individuals are influenced by higher-level components of the system (organisations, communities, society) has a causal relationship with the mental health and mental health-related behaviours of all individuals within the system.

The behaviours of individuals can be either beneficial (e.g., through social support, encouraging help seeking) or detrimental (e.g., through abuse or harassment) to the mental health of others. As such, and in line with general systems theory, individuals behaviours should be monitored and regulated from within the system to ensure that no psychological harm is done. While the role of organisations in the recreational sport system will vary by context (postulate 3), their common functions will be to prevent psychological harm, and promote mental health and wellbeing. For example, sports clubs, regional level associations, state-based sports organisations, and/or national sports organisations can all play a role in monitoring and preventing individual behaviours such as abuse that may be psychologically harmful, and incentivising compliance with behavioural guidelines and the promotion of positive behaviours that can enhance mental health and wellbeing (e.g., through evidence-based coach training or mental health programming).

## 6. Discussion

The basic premise of Postulate 1 is that one of the objectives of the recreational sports system is facilitate sports participation in a way that is psychologically safe (i.e., does no harm [57]); and can promote mental health and wellbeing. However, the extent to which this objective is recognised is somewhat ambiguous. For example, coaches of adolescent athletes have expressed some hesitancy to take on responsibility for athlete mental health due to a lack of knowledge and/or training [46]. As such, there is a question as to the extent to which all stakeholders recognise and accept this objective. Further, a second research question is whether recognition and acceptance of the objective by individuals within the system (e.g., coaches, administrators) leads to greater action or more beneficial mental health outcomes. For example, at an organisational level, is a recognition of the objective to protect and promote mental health reflected in organisational policies (e.g., mental health policies, mission statements) or practices (e.g., funding for mental health training). As a practitioner working in the area of mental health and sport, the postulate dictates that efforts be expended to raise awareness and acceptance of the objective to protect and promote mental health in recreational sports, and help to facilitate the necessary strategies that are needed to fulfil that objective.

Postulate 2 asserts that each system component (e.g., individuals, groups, organisations) will play a specialised function within that system. However, there is some ambiguity around the specific roles and functions of each system component. Defining the specific roles of each component is critical in moving forward with coordinated action. For example, what is the role of individuals (e.g., athletes, parents, siblings, coaches, and volunteers) in ensuring successful delivery of recreational sport programs, and how might this role differ from groups (e.g., teams, age groups) and organisations (e.g., clubs, leagues, governing bodies)? Further, Postulate 3 dictates that in defining these roles and functions, both expert and stakeholder input should be sought so that a robust, contextualised understanding of the system components can be articulated and that principles of coordinated action can be co-designed with individuals who represent those components. Once such roles are articulated, practitioners will be critical in facilitating uptake of those roles.

With regard to the necessary strategies to protect and promote mental health, Postulate 4 proposes that the coordinated action of system components such as governing bodies, sports organisations, and individuals is necessary to optimise mental health outcomes. A recent review into mental health policy, evidence, and practice in elite sport led the authors to conclude that:

“The evident lack of cohesion within the field results in a range of implications for both athletes, and all those working to support athlete mental health. A range of steps needs to be taken to ensure researchers, policymakers, and practitioners work cohesively”

[58]

The application of postulate 4 is therefore to work to ensure the cohesive and coordinated action of the various stakeholders within the system, including governing bodies, sports organisations, and individuals. As demonstrated by Prior et al. [58] there are also questions to be answered in regard to the current state of coordinated actions within the recreational sports system. For example, assessing or auditing the current state (e.g., policies, resources), and future needs, of a recreational sport system in regard to mental health promotion may be a first step to ensuring that comprehensive coordinated action can be facilitated in practice.

Finally, postulate 5 dictates that definitions and operationalisations of deviations from acceptable practice are observable and able to be rectified from within the system. For example, this may include the articulation of minimally acceptable standards of practice for individuals (e.g., coaches, parents) or sports organisations (e.g., policies that need to be in place, training that needs to be provided). Articulating these standards should be done in conjunction with an understanding of the roles of each system component and specify who, or which, organisation is responsible for providing, overseeing, and enforcing those minimally acceptable standards or practices. As such, there is an essential question to be answered regarding what minimally acceptable standards and practices are, as well as the processes or resources needed to meet those standards.

We anticipate that the articulation of this systems theory of mental health in recreational sport will stimulate further debate around the role of the system as a whole in the protection and promotion of mental health among all of those who are involved, with the aim of moving toward some consensus. We also anticipate that the articulation of this theory will stimulate a necessary increase in empirical and policy attention on recreational sports where the vast majority of participation in sport occurs [19]. Finally, we anticipate that the theory can be used as a foundation for the design and implementation of such an increase in empirical and, in turn, evidence-based policy work.

One limitation in this regard is that this theory is not prescriptive in nature—it does not articulate the ways in which individuals, organisations, communities or societies should act in order to prevent psychological harm or promote mental health and wellbeing. Rather, the proposed theory of mental health in recreational sport is descriptive in nature, and focusses on the ways in which major components of the system interact to influence mental health and wellbeing at an individual level. Nonetheless, in this respect the theory can inform the types of research questions posed, and can guide the major areas of consideration for mental health policies as described above. For example, policies regarding mental health in recreational sport might be well served to include actionable guidelines for all components of the sports system including individuals (e.g., athletes, parents, coaches, officials) and organisations (e.g., clubs, regional organisations, state or national organisations).

A second challenge is that the influence and function of all the components of the recreational sporting system are explicitly theorised to vary by context—which may include variations by sport and geographical location at a minimum. Nonetheless, this also presents an opportunity to generate empirical insights that can be informative for research and policy into the future. For example, the articulation of the roles and functions of individuals and organisations within the recreational sporting system, which is based on evidence and considers the context in which these roles exist (e.g., community resources, societal expectations), is a clear priority for both researchers and policy-makers. This knowledge can be operationalised in beneficial ways, including the articulation of mental health guidelines or models of psychological care that are tailored to the context in which that system is embedded. Such guidelines or models can be of tremendous use in the prevention of psychological harm and promotion of mental health and wellbeing in recreational sports.

Given the relative lack of empirical evidence, the state of mental health in recreational sports in unclear. While some of the risks of psychological harm have been well articulated [59], and there is evidence that recreational sport participation is associated with better mental health over time [60,61], there is very little evidence to support the major influences on psychological safety or mental health in recreational sporting systems. There is now a major and urgent opportunity to assess the state of mental health in recreational sport, including the key influences on individual level mental health, such as the range of potential psychological harms, and how those influences interact to harm, protect, or promote mental health. This theory can help to guide such empirical exploration.

Finally, the theory can help to guide the design and implementation of interventions that work to protect and promote mental health. A recent systematic review demonstrated that mental health interventions in recreational sport are largely ineffective [9]. One of the most evidence-based and effective programs is Ahead of the Game—a multicomponent mental health literacy and resilience program designed to protect mental health and enhance wellbeing of adolescent male sport participants [37]. This program is purposely designed to address the multiple influences on mental health in sport, with distinct program components for the athletes, coaches, the team unit, parents, and the sports club [62]. Other, similar programs which are aimed at only adolescent sport participants and thereby do not address the influence of other components of the recreational sporting system are not effective at the group level, and may only be effective for a small subsection of sport participants who start with the lowest scores on baseline measures [63]. This highlights the need to draw on theory when designing and implementing mental health programs with recreational sports in order to facilitate effective change. The focus on mental health outcomes in sport has proliferated across all sports contexts, including recreational sports. However, empirical and policy efforts in recreational sports have lacked a sound theoretical basis, and have been ad hoc in nature. To move forward, theoretical models or frameworks of mental health in sport are urgently needed, and these should be specific to the needs of recreational sports.

## 7. Conclusions

We have defined the recreational sports system and have provided a basis for a theory of mental health in recreational sport in line with general systems theory [20]. Through the articulation of five postulates we have articulated the core assumptions of the theory of mental health in recreational sport. We have also outlined the ways in which the subcomponents of the system interact, their potential influence on mental health in recreational sports, and the ways in which they may be regulated. Thus, we have provided a theoretical foundation upon which empirical research and mental health related policies can be based, as well as the design and implementation of effective programs to protect and promote the mental health of all those involved in recreational sports.

## Data Availability

Not applicable.
